# Strategies to increase vitamin C in plants: from plant defense perspective to food biofortification

**DOI:** 10.3389/fpls.2013.00152

**Published:** 2013-05-22

**Authors:** Vittoria Locato, Sara Cimini, Laura De Gara

**Affiliations:** Laboratory of Plant Biochemistry and Food Sciences – Università Campus Bio-MedicoRome, Italy

**Keywords:** vitamin C, crop, food nutritional value, bio-engineering, QTL analysis

## Abstract

Vitamin C participates in several physiological processes, among others, immune stimulation, synthesis of collagen, hormones, neurotransmitters, and iron absorption. Severe deficiency leads to scurvy, whereas a limited vitamin C intake causes general symptoms, such as increased susceptibility to infections, fatigue, insomnia, and weight loss. Surprisingly vitamin C deficiencies are spread in both developing and developed countries, with the latter actually trying to overcome this lack through dietary supplements and food fortification. Therefore new strategies aimed to increase vitamin C in food plants would be of interest to improve human health. Interestingly, plants are not only living bioreactors for vitamin C production in optimal growing conditions, but also they can increase their vitamin C content as consequence of stress conditions. An overview of the different approaches aimed at increasing vitamin C level in plant food is given. They include genotype selection by “classical” breeding, bio-engineering and changes of the agronomic conditions, on the basis of the emerging concepts that plant can enhance vitamin C synthesis as part of defense responses.

## INTRODUCTION

**FIGURE 1:**
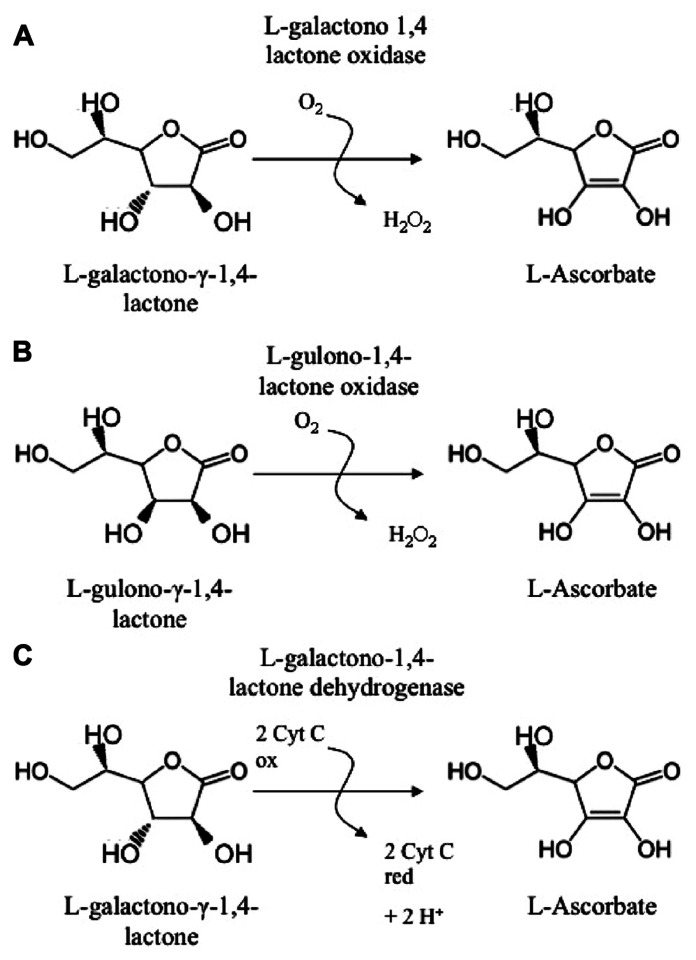
**Last enzymes in ASC biosynthetic pathways**. **(A)** Pathway in protists and fungi; **(B)** Animal pathway; **(C)** Main pathway in higher plants.

Ascorbate (ASC) is a major soluble redox molecule with pivotal roles in allowing several metabolic pathways to work properly. ASC regenerates other metabolites, among which tocopherols, from oxidative damages and protects the catalytic site of a number of enzymes (e.g., hydroxylases) from irreversible oxidation, possibly caused by reactive oxygen species (ROS) in both animal and plant cells. It can be used as substrate or enzyme cofactor in various biological reactions (Lodge, 2008; De Gara et al., 2010). ASC is synthesized by fungi, protozoa, plants, and animals, even if by means of different biosynthetic pathways (Bleeg and Christensen, 1982; Banhegyi et al., 1997; Wheeler et al., 1998; Logan et al., 2007). For few animal species, among which guinea pig, some birds, humans, and primates in general, ASC is a vitamin (vitamin C), since during their evolution, they have lost the capability to synthesize it. In human this was caused by the loss of functionality of gulono-1,4 γ-lactone oxidase (GuLO), the enzyme catalyzing the last step of animal ASC biosynthesis (Nishikimi et al., 1992, 1994; **Figure 1**). Although ASC is considered essential for aerobic life (De Gara et al., 2010; Gest et al., 2013), it is worth noting that in fungi, protozoa, and animals the last reaction of its biosynthesis also produces hydrogen peroxide (H_2_O_2_), a putative toxic species (Banhegyi et al., 1997; **Figure 1**). Therefore, in animals consuming foods that ensure sufficient ASC intake, the loss of ASC biosynthetic capability may be done an evolutionary acquisition leading to an ameliorated control of redox homeostasis in the cells of these organisms. It is interesting to notice that plants, which produce a great amount of ASC in almost all their tissues (ASC reaches several tens of millimolar concentrations in green tissues), have evolved a synthesizing pathway with the last step catalyzed by a dehydrogenase (L-Galactono-1,4-γ-lactone dehydrogenase GaLDH) that does not produce H_2_O_2_ (**Figure 1**).

Plant-derived food are the main dietary source for vitamin C (**Table 1**). Vitamin C is also present in some meats, such as cow liver (liver and kidney are ASC synthesizing organs in animals, Kanfer et al., 1959), but they are irrelevant in supplying vitamin C because of their limited use in human nutrition and the consistent lost of ASC content caused by food processing, mainly due to ASC thermal instability (Munyaka et al., 2010).

**Table 1:** Vitamin C content in plant edible organs.

Fruit and Vegetables	mg Vit C/100 g FW
Guava	243
Currant	200
Pepper	146
Rocket	110
Turnip Tops	110
Kiwi	85
Brussels Sprouts	81
Broccoli	77
Papaya	60
Cauliflower	59
Strawberry	54
Spinach	54
Clementine	54
Orange	50
Lemon	50
Tangerine	42
Grape Fruit	40
Endive	35
Broad Bean	33
Celery	32
Tomato	23
Melon	22
Radish	18
Lettuce	16
Banana	16
Potatoes	15
Soya Bean Sprout	13
Fennel	12
Apple	8
Carrot	4
Pear	4
Peach	4

*A selection of plants of interest for human nutrition has been obtained from a database on food chemical composition (http://www.ieo.it/bda2008/homepage.aspx). Only the vitamin C content of the edible organs has been reported.*

Severe vitamin C deficiency causes scurvy, a disease discovered in the sailors of 15th and 16th century that could not consume fresh plant-derived food for months (Baron, 2009). Scurvy has been considered one of the most important disease derived from nutrient deficiency in the history of humanity (Magiorkinis et al., 2011). Scurvy symptoms consists in generalized edema, skin hemorrhages, swollen, bleeding gum and, if prolonged, can cause death (Magiorkinis et al., 2011). It is generally accepted that it is due to an impairment of collagen formation (Peterkofsky, 1991). Indeed ASC participates to collagen cross-linking reactions as cofactor of prolyl hydroxilases. The role of ASC in these reactions is to maintain the iron present into the enzymatic catalytic site in the reduced state and thus converting back the inactivated form of the enzymes into the active one (Gorres and Raines, 2010). Today scurvy is rare in developed as well as developing countries, since it requires a severe and prolonged deficiency in vitamin C in order to become evident; however, recent epidemiological studies underline that even in western populations sub-optimal vitamin C intake is widespread (Troesch et al., 2012).

Recommended dietary allowance (RDA) for vitamin C is a controversial matter, since different countries provide different advice; for example RDAs for adult men are 40 mg/day in UK; 90 mg/day in USA; 100 mg/day in Germany; 70 mg/day in Netherland (Troesch et al., 2012). Moreover, in order to enhance health benefits due to vitamin C intake, the scientific community is suggesting to increase its RDA to 200 mg/day (Frei et al., 2012). Epidemiologic studies have actually revealed that ASC intake over the current RDA has a significant impact in reducing the risk of diseases such as respiratory tract infections, cardio-vascular diseases and cancer (Schlueter and Johnston, 2011). With the exception of special population groups, as for example people suffering for kidney stones (whose formation could be promoted by oxalate, a catabolic derivate of ASC in mammals; Linster and Van Schaftingen, 2007), adverse effects caused by vitamin C over-ingestion, such as diarrhea, only occurs when the intake overcomes 2000 mg/day (Schlueter and Johnston, 2011). Moreover, pro-oxidant effects of vitamin C was only reported for daily intake higher than 500 mg (Podmore et al., 1998).

**FIGURE 2:**
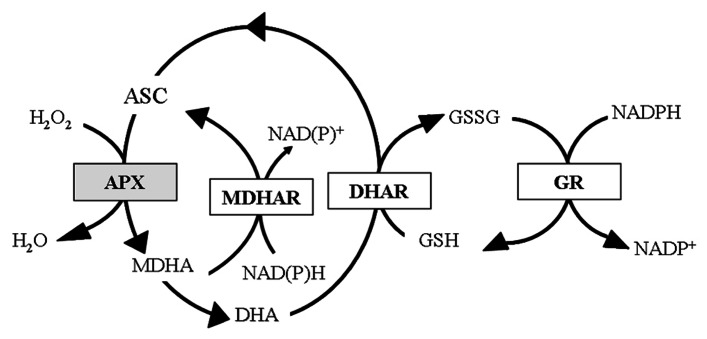
**Foyer- Halliwell- Asada cycle**. Enzymes and intermediates of the cycle (also known as ASC-GSH cycle) are reported. In white boxes the enzymes active in both animal and plant cells; in gray box the enzyme exclusively presents in plant cells. APX, ascorbate peroxidase; MDHAR, monodehydroascorbate reductase; DHAR, dehydroascorbate reductase; GR, glutathione reductase.

In human, vitamin C uptake is controlled by specific Na-dependent active transporters probably present in all the cells accumulating ASC (Savini et al., 2008). In animal tissues, the oxidized form of vitamin C, dehydroascorbate (DHA), is less efficiently taken up on glucose transporters. Within cells DHA is reduced back to ASC (the active form of the vitamin C) by enzymes using glutathione (GSH) and pyridine nucleotides as electron donor (Rumsey et al., 1997; Linster and Van Schaftingen, 2007; **Figure 2**). According to literature data, 200 mg/day is the intake of vitamin C that leads to the saturation of renal clearance for reabsorption of vitamin C, since a dose-dependent vitamin C release is observed in urine starting from an intake higher that this dose (Levine et al., 1996, 2001). In this perspective all the current RDAs for vitamin C (see above), assessed for avoiding scurvy and mild deficiency, are starting to be considered suboptimal in regards to the potential health benefits triggered by this vitamin (Frei et al., 2012).

In developed countries vitamin C supplementation is largely adopted especially for preventing/reducing cold related diseases. Actually the industrial production of vitamin C represents a low efficient and expensive technology (Hancock and Viola, 2002). Recently, it has also been demonstrated that vitamin C from plant-derived food (i.e., kiwifruit) is more bio-available than the chemically synthesize or purified molecule used in supplementation (Vissers et al., 2011). This could be the consequence of the presence in food matrixes of plant origin of several molecules with antioxidant/redox properties, which can have a synergic effects with ASC or be able to preserve vitamin C in its active reduced state (Villanueva and Kross, 2012). The possibility that other biological molecules could stabilize ASC is supported by DHA/ASC redox potential (estimated around as 90 mV; Noctor, 2006) that makes ASC a good reductant and, at the same time, its oxidized form DHA reducible in cellular metabolic conditions (Szarka et al., 2012).

Another aspect that makes interesting to increase vitamin C level in the edible plant tissues is the fact that this metabolite improves the post-harvesting properties of several vegetables. Indeed, its addition to several food matrix is used for extending their shelf life, as well as for improving specific technological properties, such as the kneading of wheat flour and dough rheological properties (Paradiso et al., 2006).

On these bases, ASC bio-fortification of the plants utilized for food production is becoming an important nutritional claims also for having promising technological implications. The plethora of roles played by ASC in plant metabolism increases the complexity of this goal. Here an overview of the results obtained in vitamin C bio-fortification is given with particular attention to the results obtained on crops and on the reasons why vitamin C bio-fortification still remains an ambitious target.

## STRATEGY OF BIOFORTIFICATION

### EFFECT OF GENOTYPE AND AGRONOMIC PROCEDURES

**FIGURE 3:**
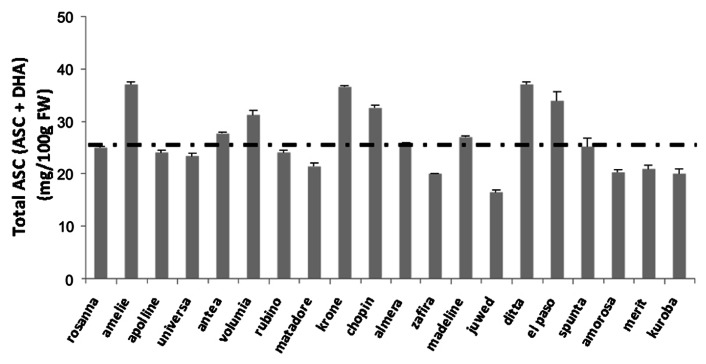
**Variability of ASC content in early potato tubers grown in the same agronomic conditions**. The content of vitamin C in 20 cultivars of early potato grown in the same experimental field in Apulia Region (southern Italy) is reported. The values are the mean of six different experiments ± standard error. Dotted line represents the value reported in official database as the standard value for early potato tuber cultivated in Italy (http://www.ieo.it/bda2008/homepage.aspx).

It is well known that different crop varieties produce and store different amounts of vitamin C in their tissues. Maize heterotic F1 hybrid (B73xMo17) have higher ASC biosynthetic capability and activities of ASC-GSH cycle enzymes, in comparison with the parental lines B73 and Mo17 (De Gara et al., 2000). In several other crops the effects of genotype on vitamin C levels of edible tissues have been reported (see as few examples Kalt et al., 1999; Kafkas et al., 2006). Variability in ASC content has been deeply studied in *Solanum tuberosum*. In spite of potato tubers storing a moderate amount of vitamin C (8–36 mg/100 g fresh weight) in comparison with other plant-derived foods (**Table 1**), the possibility to improve its level in the tubers is of great interest. Indeed this species is relevant in supplying vitamin C, in particular in the developing countries where potatoes make up a large part of a subsistence diet. The first evidence for the genetic basis of ASC variability as well as the possibility of breeding *S*. *tuberosum* for increasing its vitamin C content, have been reported more than 30 year ago (Augustin et al., 1978). **Figure 3** reports the contents of vitamin C in 20 cultivars of early potato growth in the same experimental field in Apulia Region (southern Italy). The variation in the content of this nutrient is evident, as well as the deviation from the mean value considered as standard for the same kind of vegetables grown in Italy (indicated as dotted line in **Figure 3**; Buono et al., 2005; Buono et al., 2009). A multi – year study using 75 genotypes from 12 North American potato – breeding programs suggests that most of the tested genotypes produces different amount of vitamin C in response to different growth conditions occurring in the same environment over time. However, few clones with a stable and high capability of storing vitamin C have been selected and suggested as putative genotypes of interest for large scale production of potatoes with enriched levels of vitamin C (Love et al., 2004).

Correlations between ASC contents and environmental conditions have been also taken into consideration both in model and crop plants. The intra-species variability of vitamin C or other antioxidants have often been considered relevant for explaining the differences among cultivars in sensitivity to a plethora of biotic and abiotic environmental stresses. Literature data suggest that tolerance to stress also correlates with the capability of increasing ASC biosynthesis or the activity of ASC – related enzymes, when plants are exposed to unfavorable environmental conditions (Gill and Tuteja, 2010; Wang et al., 2012). Consistently, mutants with reduced level of vitamin C, i.e., *Arabidopsis* vtc mutants, have been selected for their sensitivity to specific stress conditions (Smirnoff and Wheeler, 2000). An increase in ASC content is also induced by iron deficiency in sugar beet roots, where a 20-fold increase in the activity of root ferric chelate reductase was accompanied by a twofold increase in vitamin C level (Zaharieva and Abadia, 2003).

The effects of water or salt stress have been studied in several crops (Mittova et al., 2004; Hakeem et al., 2012; Turan and Tripathy, 2013). An increase in salt (NaCl) from 3 to 6 dSm in soilless systems induces a significant rise in the level of ASC, α-tocopherol and dry matter of “cherry” tomato fruits (Serio et al., 2004). Interestingly, irrigation with saline water is commonly used in Southern Italy with the aim at increasing flavor of tomato fruits and other vegetables, NaCl is also supplied for the production of early tomatoes in soilless systems in Northern Europe (Adams, 1991; Raffo et al., 2002).

Treatments with molecules involved in the stress signaling pathways, such as jasmonates, are able to induce a twofold increase in ASC content by altering the expression of genes coding for several enzymes involved in its metabolism (Sasaki-Sekimoto et al., 2005). A relevant increase in ASC content has also been induced both in model and crop plants by exogenous supply of L-galactono 1,4-γ-lactone (GaL), the last precursor of ASC biosynthesis (**Figure 1**). In *Lupinus albus* seedlings a dose – dependent increase of ASC contents in the vegetative tissues is induced by supplying GaL in hydroponic growth medium. This increase also correlates with increase in seedling rate growth due to the ASC-dependent stimulation of both cell division and cell elongation (Arrigoni et al., 1997). GaL exogenous treatments also increase ASC content in wheat leaves and kernels. Interestingly, this ASC increase induces a delay in the activation of programmed cell death, a process typically occurring at the end of the storing process in cereal endosperm cells. The ASC-enriched kernels also have an increased weight and protein content, probably due to the extension of the filling phase (Paradiso et al., 2012). However, although GaL supply could be an efficient strategy for vitamin C biofortification, its feasibility on large scale is discouraged by the high cost of the treatments.

### BIO-ENGINEERING OF ASC BIOSYNTHESIS

**FIGURE 4:**
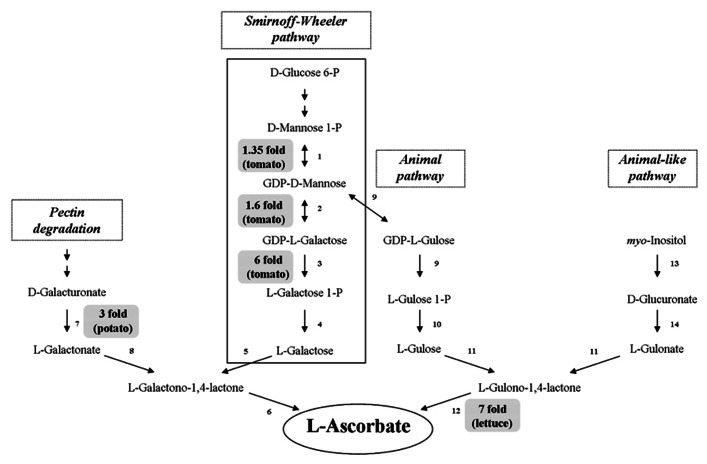
**ASC biosynthetic routes and ASC increases in bio-engineered crops**. The ASC biosynthetic pathways active in plant tissues are reported. In gray boxes the highest increases obtained by overexpressing the corresponding enzyme are indicated. Enzymes: 1 GDP-Mannose pyrophosphorylase (GMPase), 2 GDP-Mannose epimerase (GME), 3 GDP-Galactose phosphorylase (GGP), 4 L-Galactose 1P phosphatase (GPP), 5 L-Galactose dehydrogenase, 6 L-Galactono-1,4-γ-lactone dehydrogenase (GaLDH), 7 Galacturonate reductase (GalUR), 8 aldono-lactonase, 9 phosphodiesterase, 10 sugar phosphatase, 11 L-gulose dehydrogenase, 12 L-Gulono-1,4-γ-lactone oxidase (GuLO), 13 Myo-inositol oxygenase (MIOX), 14 D-Glucuronate reductase.

As virtually for all metabolites, ASC accumulation can be achieved in plants by manipulating its metabolism at biosynthetic, catabolic or recycling level. Indeed transgenic crops with increased ASC level have been obtained by increasing the expression of its biosynthetic or recycle enzymes. It is generally accepted that ASC *de novo* synthesis occurs in higher plants mainly through the Smirnoff–Wheeler pathway (S-W; Wheeler et al., 1998; **Figure 4**). This is supported by the fact that all the identified *Arabidopsis thaliana* mutants, which are partially deficient in ASC (vtc1, 2, 3, 4, 5), are impaired in the expression of enzymes involved in this pathway. Moreover, mutants completely lacking the ability to produce ASC through this pathway are lethal (Conklin et al., 2000; Dowdle et al., 2007). The S-W route uses mannose and galactose as main intermediates and it shares these metabolites with pathways leading to the synthesis of glycoproteins and cell wall polysaccharides (Lukowitz et al., 2001; Reuhs et al., 2004). Therefore, alterations in the metabolic fluxes toward one of these pathways might also affect the availability of intermediates for the other correlated pathways, with relevant consequences for plant development and fitness. As previously mentioned, the direct precursor of ASC in S-W pathway is GaL which is converted to ASC by a dehydrogenase localized in the inner mitochondrial membrane (GaLDH, step number 6 in **Figure 4**). GaLDH seems to be part of the respiratory complex I and requires oxidized cytochrome C as electron acceptor (Bartoli et al., 2000; Millar et al., 2003). This tight link between ASC biosynthesis and respiratory electron chain makes ASC synthesis in plant cells strongly sensitive to certain stress conditions that cause impairment in electron flux through respiratory complexes (Bell et al., 1971; Millar et al., 2003; Vacca et al., 2004). A strict correlation between mitochondrial electron flow and ASC biosynthesis seems also to occur in some climacteric fruits. In tomato the increase in ASC level, occurring during fruit ripening after breaker stage, might be correlated with an increase in respiration rate (Ioannidi et al., 2009). Ethylene itself seems to control ASC biosynthesis. In tomato fruits ethylene treatment stimulates the expression of L-Galactose 1P phosphatase (GPP; step number 4 in **Figure 4**); while in *Arabidopsis* the overexpression of the ethylene responsive transcription factor ERF98 increases ASC biosynthesis probably through the ERF98 interaction with the promoter of GDP-mannose pyrophosphorylase (GMPase, step number 1 in **Figure 4**; Ioannidi et al., 2009; Zhang et al., 2012). On the other hand, in climacteric fruits ASC is also responsive for ethylene production, being co-factor of 1-aminocyclopropane-1carboxylic acid oxidase, the last enzyme of ethylene biosynthesis (Ververidis et al., 1992; Liu et al., 1999). In kiwifruit, another climacteric fruits, the highest ASC level occurs in an early stage of development and thus seems to be independent on ethylene production (Li et al., 2010a). These findings underline the complexity of a network of events that has different peculiarities depending on the species.

The overexpression of enzymes involved in S-W pathway have increased vitamin C level from 1.2- up to 6-fold in the edible parts of plants, such as tomato, potato, and strawberry (**Figure 4**). The highest increase has been obtained by Bulley et al. (2012) in transgenic tomato overexpressing GDP-galactose phosphorylase (GGP, step number 3 in **Figure 4**). These transgenic plants also have smaller fruits which were seedless or provided with nonviable seeds. In tomato and strawberry fruits GGP overexpression induces polyphenol levels higher than in wild-type (2–0.5-fold, respectively; Bulley et al., 2012). The overexpression of genes responsible for the biosynthesis of specific phenolic compounds has often been reported to induce seedless fruit production (Ingrosso et al., 2011 and references wherein). Moreover, it is well known that changes in ASC content, redox state and related redox enzymes characterize the different phases of seed maturation (Arrigoni et al., 1992; De Gara et al., 2003). Therefore, alterations in both polyphenol and ASC levels might contribute to the observed impairment in seed development and viability. Interestingly, ASC bioavailability seems to be increased by high polyphenol concentration in plant-derived food; this feature further increases the putative nutritional value of these engineered crops (Vissers et al., 2013).

Tomato plants overexpressing GDP mannose epimerase (GME, step number 2 in **Figure 4**) or GDP-GMPase, (step number 1 in **Figure 4**) have been also obtained with a modest increase in ASC accumulation in ripe fruits (up to 1.6–1.35-fold compared to wild-type plants, respectively; Zhang et al., 2010; Cronje et al., 2012). GME mutants also show enhanced tolerance to oxidative stress (Zhang et al., 2010).

Another success in term of vitamin C bio-fortification has been obtained by expressing rat GuLO (step number 12 in **Figure 4**) in lettuce, where up to a sevenfold increase of ASC level has been obtained (Jain and Nessler, 2000). The high increase in ASC biosynthesis observed in this plant could also be due to the very low level of ASC present in wild-type leaves (about 4 mg/100 g FW).

Surprisingly, no positive evidence of increasing ASC content by overexpressing GaLDH in crops have been reported in literature, at least to our knowledge. GaLDH overexpression only gives a positive effect on ASC biosynthesis in cultured tobacco cells (Tokunaga et al., 2005). The failure in obtaining an increase in ASC level by overexpressing the last enzyme of its biosynthetic pathway could be correlated by the presence of feedback control of ASC toward its *de novo* synthesis (Mieda et al., 2004; Mellidou et al., 2012a). When ASC reaches a threshold value a feedback control is activated by inhibiting one of the initial steps of the pathway. Therefore the catalytic activity of the last enzyme strongly depends on the availability of its substrate (De Gara et al., 1989; Mieda et al., 2004; Mellidou et al., 2012a).

Intermediates not present in the S-W pathway, such as D-galacturonate and myo-inositol, have been also identified as ASC precursors in plants; thus suggesting that alternative pathways can be utilized for the vitamin production. Indeed cell wall degradation leads to galacturonate release, thus providing this intermediate for ASC biosynthesis. This minor route has been suggested to become relevant during tomato ripening, when pectine degradation, responsible for fruit softening, increases the availability of alternative ASC precursor (Badejo et al., 2012). The increase in pectine degradation by pectinesterase or polygalacturonase overexpression probably does not represent a feasible strategy to increase ASC level, since it might decrease fruit shelf life. In this perspective the extension of the shelf life of tomato fruits achieved by the down-regulation of polygalacturonases (Smith et al., 1988) could also decrease the ASC accumulation in the mature fruits of the engineered plants. On the other hand, it has been reported that ASC itself acts on fruit softening by promoting pectine breakdown through a non-enzymatic mechanism: in the presence of Cu^2^^+^ and H_2_O_2_ in cell wall, ASC induces hydroxyl radical formation responsible for direct polysaccharides scission (Fry et al., 2001; Dumville and Fry, 2003). Therefore the relation between ASC levels and fruits firmness is another complex aspect that merits to be better investigated even in order to select the best strategies for obtaining fruits enriched in vitamin C by means of bio-engineering approaches.

Galacturonate reductase (GalUR step number 7 in **Figure 4**) expression positively correlated with ASC content in strawberry (Agius et al., 2003). GalUR overexpression has been performed leading to an increase in ASC accumulation up to threefold in potato tubers (Hemavathi et al., 2009). Myo-inositol, a compound involved in the biosynthesis of signaling molecules, can also generate D- glucuronate, which is then transformed into gulono-1,4-γ-lactone as direct ASC precursor, thus suggesting the existence of an animal-like pathway for ASC production also in plant (Lorence et al., 2004). It has been also hypothesized that the enzyme of S-W route GPP catalyses also myo-inositol production by de-phosphorylation of myo-inositol phosphates feeding ASC production in plants through both pathways (Torabinejad et al., 2009). Myo-inositol oxygenase (MIOX; step 13 in **Figure 4**) overexpression has successfully been tested for increasing ASC in the model plant *Arabidopsis thaliana* (up to threefold), but not in tomato (Lorence et al., 2004; Cronje et al., 2012). This could be due to a species – specific capability to use alternative routes to synthesize ASC and further underlines the peculiarity of each plant species in ASC accumulation.

### BIO-ENGINEERING FOR INCREASING ASC RECYCLE

As in animals, also in plants the stable oxidized form of ASC is DHA even if ASC undergoes to an univalent oxidation giving radical monodehydroascorbate (MDHA). Therefore spontaneous MDHA dismutation produces DHA. MDHA and DHA regenerate ASC through the recycling reactions catalyzed by MDHAR and DHAR in the Foyer-Halliwell-Asada cycle (Foyer and Halliwell, 1976; Foyer and Noctor, 2011; **Figure 2**). This cycle is ubiquitous in plants and different isoforms of its enzymes are present in almost all subcellular compartments (Locato et al., 2009). A high efficiency in the ASC regeneration from its oxidized forms has been proposed as the mechanism allowing the mature fruits of tomato cultivar Santorini to store higher levels of ASC compared to those present in other cultivars (Mellidou et al., 2012a). However, DHAR overexpression induces a modest increase in ASC accumulation in maize kernels and potato tubers (Chen et al., 2003; Qin et al., 2011). On the other hand, data on the effects caused by MDHAR overexpression are contradictory. In tobacco the expression of an *Arabidopsis* peroxisomal isoform of MDHAR targeted to cytosol induces a 2.2-fold increase in ASC level (Eltayeb et al., 2007); while tomato transgenic lines, obtained by overexpressing the tomato MDHAR3 targeted to cytosol and peroxisomes, have shown unchanged or even decreased ASC accumulation in fruits and leaves, respectively. In the same study, transgenic lines with silenced MDHAR3 showed significant ASC increase in both fruits and leaves (Gest et al., 2012). The differences in the capability to accumulate ASC between the two kinds of transgenic plants were enhanced by high light exposure (Gest et al., 2012). It is known that light exposure increased ASC production in plants (Smirnoff, 2000; Tabata et al., 2002). Recently, it has been reported that a light-dependent increase in ASC occurs in both fruits and leaves being stronger in the latter (Li et al., 2010b; Massot et al., 2012). This is consistent with the pivotal role of ASC in the chloroplastic photo-protecting mechanisms: it is cofactor of violaxanthine de-epoxidase, an enzymes involved in the xanthophyll cycle, and the major player of the water–water cycle, two pathways preserving photosynthetic components by the photo-oxidation due to high irradiance-dependent ROS release (Eskling et al., 1997; Asada, 2000). ASC also acts as electron donor of PSII when the oxygen evolving system is lost (Tóth et al., 2009, 2011). The involvement of ASC in redox reaction aimed at protecting photosynthetic functionality may explain why the overexpression of the ASC recycling enzymes generally induces an increase in plant tolerance toward a number of stresses, such as chilling, salt, ozone even when it does not substantially affect ASC levels in plant tissues (Eltayeb et al., 2007; Stevens et al., 2008; Li et al., 2010c).

Interestingly, the enhancement of ASC level by overexpressing its recycle enzymes has been suggested as a good strategy for extending shelf life of edible plants that can be stored at low temperature as in the case of apple fruits. The increased capability of ASC recycle makes these fruits more tolerant to cold stress, since ASC oxidation to DHA has been suggested to be responsible for the flesh browning during the long storage period (6 months; Mellidou et al., 2012b).

When DHA is not converted back to ASC an irreversible loss of the vitamin occurs. It has been reported that in plant cells DHA catabolism irreversibly converts this molecule to oxalate and threarate (Green and Fry, 2005). Even if the involvement of enzymes in DHA catabolism has been hypothesized, oxalate and threarate production from DHA catabolism also occurs spontaneously *in vitro*. This makes the control of this process by bio-engineering not viable (Parsons et al., 2011).

## ASC OXIDATION, A NECESSARY LOST FOR PLANT METABOLISM

Enzymatic ASC oxidation mainly occurs in plants through the reactions catalyzed by ascorbate oxidase (AOX) and ASC peroxidase (APX), two typical plant enzymes. AOX is an apoplastic enzyme involved in cell elongation (Takahama and Oniki, 1994). The down-regulation of this enzyme causes a shift in the apoplastic ASC pool toward its reduced state; it also increases plant yield during water deficit, through a carbon flux re-allocation, but does not determine a significant ASC increase in the investigated tissues (Garchery et al., 2013).

APX down-regulation is not a feasibly strategy for preserving ASC in plant, since this enzyme is a key player in many plant defense responses. A decrease in its activity might enhance plant susceptibility to stress (Örvar and Ellis, 1997; de Pinto et al., 2006). Indeed, the appearance of various APX isoforms during evolution can be considered a specific acquisition for promoting survival of these sessile organisms by using a molecule that plants can produce by themselves (Ishikawa and Shigeoka, 2008). APX uses ASC as electron donor to scavenge H_2_O_2_ normally produced in aerobic metabolism and over-produced during abiotic and biotic stresses (Karpinski et al., 1997; Mittler et al., 1999; Paradiso et al., 2008; De Gara et al., 2010; **Figure 2**). It has been suggested that transcriptional and post-transcriptional regulation of APX is a signaling strategy able to finely regulate the H_2_O_2_ level into the cell, switching the role of this molecule from toxic compound to signaling molecule (de Pinto et al., 2012). Actually, being the only ROS able to cross cell membranes, H_2_O_2_ can work as messenger in the transduction pathway activated as consequence of different stimuli (Foyer and Noctor, 2005; Bienert et al., 2007). Indeed H_2_O_2_ has been supposed to regulate gene expression during plant defense response playing a major role in tolerance acquisition against stress (Miller et al., 2008). For example, in thermal acclimation, H_2_O_2_ is reported to regulate the expression of heat shock proteins and of a thermostable APX isoenzyme (Banzet et al., 1998; Lee et al., 2000; Suzuki and Mittler, 2006; Volkov et al., 2006).

A general enhancement of antioxidant systems are involved in plant acclimation to stress. This has relevant implications in post-harvest procedures, since post-harvest controlled stress, such as moderate temperature, are able to increase antioxidant shield in plant tissues thus improving food quality during storage (Cisneros-Zevallos, 2003).

## A SYSTEMIC APPROACH

As emerging from data reported above, ASC is involved in a wide net of metabolic reactions controlling growth and development as well as stress responses of plants (as reviewed by Foyer and Noctor, 2011). Indeed a plethora of different reactions depend on ASC and affect its level (from ROS removal to the synthesis of secondary metabolites and phyto-hormones, or to prolyl hydroxylation). This is probably why bio-engineering of a single gene involved in ASC biosynthesis or recycle often led to unsatisfactory results (see above). Actually, ASC level in plant organs and tissues can be considered a quantitative complex trait.

For this reason, in order to obtain plant-derived foods with a consistent enrichment in vitamin C, systemic approaches have been recently used. At this purpose a quantitative trait loci (QTL) analysis have been implemented in order to identify polygenic traits able to enhance ASC in edible crops. It is expected that these findings can ameliorate breeding strategies for increasing nutritional value of plant-derived foods.

Tomato has been mainly investigated, as a model crop (Stevens et al., 2007). A number of tomato wild accessions accumulating higher amounts of ASC (up to fivefold) than the cultivated lines have been identified (Di Matteo et al., 2010). An introgression line (IL) has been obtained by using the QTL identified in a wild progenitor and correlated to high ASC accumulation in mature tomato fruits. Di Matteo et al. (2010) demonstrated that the rise of ASC occurring during ripening in this IL depends on an increased flux of ASC precursors not involved in the S-W pathway. In particular, pectine degradation seems to be pivotal for feeding ASC *de novo* synthesis during tomato fruit ripening.

It is known that domestication often caused the loss of characters able to promote plant fitness and competitiveness in natural environment. In this perspective, it is possible that human selection on plants has caused a reduction in the synthesis/storage of precious metabolites. This could be occurred with vitamin C during tomato domestication. Indeed tomato varieties have been selected in the past for high yield and it probably caused ASC loss by oxidation. As already discussed, ASC oxidation promotes plant cell elongation (Takahama and Oniki, 1994; Fry et al., 2001). Moreover, the selection of tomato cultivars having prolonged fruit shelf life, and therefore prolonged flesh firmness, might lead to the selection of lines having low ASC as a consequence of a reduced or delayed pectine degradation which is responsible for fruit softening (Dumville and Fry, 2003).

However, different species can require different strategies for optimizing the post-harvest properties of their edible organs. In apples and pears the ASC level has been reported to be correlated with post-harvest quality (Veltman et al., 2000; Davey et al., 2007). In apple fruits, ASC level depends on harvest time and, as a consequence, it affects susceptibility to phytopathogens during post-harvest storage (Davey and Keulemans, 2004; Davey et al., 2007). Co-localized QTL for wound-related flesh browning and DHA content were recently identified in apple (Davey et al., 2006). ASC oxidation occurring during storage conditions has been also hypothesized to be mainly involved in post-harvest diseases of pear (Cascia et al., 2013). QTL analysis have recently suggested that candidate genes regulating ASC level and post-harvest quality in apple flesh are a paralog of GGP and MDHAR3, respectively (Mellidou et al., 2012b). An allele of MDHAR has been also proposed as major candidate gene for high ASC level in tomato fruit (Stevens et al., 2008); whereas in strawberry candidate genes for stable QLT correlated to high ASC have identified in alternative biosynthesis pathways, such as GalUR e MIOX (Zorrilla-Fontanesi et al., 2011). All these findings are supported by the role of high levels of ASC and related redox enzymes in protecting plant from stress conditions. Indeed, ASC production is enhanced by several injuries. It is in fact reported that jasmonate and its derivates produced in plant as consequence of wounding, promptly induces ASC biosynthesis (Suza et al., 2010). Consistently *Arabidopsis* vtc mutants, containing about 30% of ASC compared to wt, were identified for their increased susceptibility to ozone and then characterized for their slow growth phenotype (Conklin et al., 1996; Veljovic-Jovanovic et al., 2001). Moreover, pathogenesis related genes were up-regulated in vtc1, thus suggesting an impairment in the defense responses of this ASC-deficient mutant (Pastori et al., 2003; Pavet et al., 2005).

Another omic approach that has recently given information on metabolic networks responsive for ASC accumulation in fruit is trascriptomic analysis. Such analysis has been carried on in tomato IL showing reduced fruit ASC accumulation in comparison with its cultivated parental line. The main differences between the two lines have been identified in the steady state of mRNA related to oxidative and antioxidant pathways. In particular, this tomato IL showed an accelerated oxidative metabolism and decreased antioxidant systems compared to the parental line (Di Matteo et al., 2012). The accelerated oxidative metabolism could explain the low ASC amount in tomato IL by a reduction of sugar flux toward ASC biosynthesis. It has been suggested that ASC plays a protective role in climacteric fruits contrasting ROS rise during ripening (Jimenez et al., 2002). Therefore in the mentioned tomato IL the increased oxidative metabolism probably increases ROS, thus causing a further ASC consumption.

## CONCLUSION

As emerging above, so far scientific studies have failed in identifying a single master regulator responsible for ASC accumulation in plant -derived foods. This makes vitamin C bio-fortification a real challenge of plant science research. It is clear that ASC level of plant edible tissues depends on several cross-talking factors acting at different physiological levels. Within cells, competition for hexose fluxes between vitamin C biosynthesis and other metabolic pathways, as well as the balance between ASC consuming and recycling reactions are pivotal for ASC storing. This is further complicated by the fact that ASC is used in almost all cellular compartments which are characterized by the presence of diversely regulated isoenzymes of the Foyer-Halliwell-Asada cycle and of different ASC-utilizing enzymes. At organ level, the fluxes, between source and sink tissues, of ASC precursors and ASC itself could be a critical aspect for increasing ASC level in certain tissues or organs. These fluxes are developmentally regulated but they can also be altered by specific stresses or environmental conditions that diversely affect various organs of the plant. Therefore the strategies adopted to increase ASC in plant edible tissues or organs have to take into account all these considerations in order to obtain plants with an increased nutritional value and with the opportune productivity and resistance against adverse environmental conditions.

**FIGURE 5:**
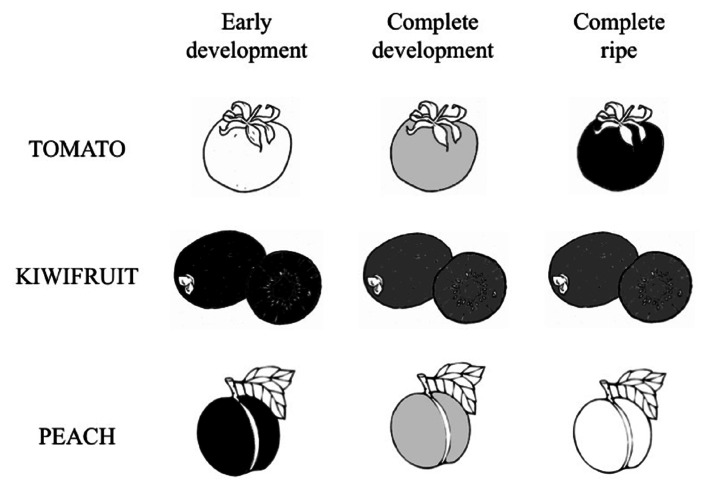
**Three models of ASC accumulation trend in different climacteric fruits**. Three illustrative development and ripening phases of different fruits. White, gray and black colors correspond to the lowest, intermediate and the highest ASC content, respectively. Since the timing of the diverse stages, as well as the vitamin C levels, are quite different, depending on several factors (see text for more details), a simplification of ASC changes over fruit maturation is given on the basis of data reported in Ioannidi et al., 2009 for tomato; Imai et al., 2009 for peach, Li et al., 2010a for kiwifruit.

Since fruits can be considered the best dietary sources for vitamin C being consumed raw, they are the main target of vitamin C bio-fortification. In this perspective, the identification of the most efficient strategy for increasing vitamin C in fruits is further complicated by the variety of ASC accumulation trends showed during ripening of fruits from different species (**Figure 5**). In particular, depending on the specie, ASC level is reported to decrease during fruit ripening (i.e., peach; Imai et al., 2009), to remain almost constant during fruit ripening after reaching a maximum level during early fruit development (i.e., kiwifruit; Li et al., 2010a) or increasing during ripening (i.e., tomato, Ioannidi et al., 2009). These trends can show further intra-species variations at least in terms of the timing of different development and ripening stages as well as they can be affected by agronomic and environmental conditions. All these aspects make clear why ASC manipulation of biosynthesis or recycle level not always has led to a consistent vitamin C bio-fortification (Zhang et al., 2010; Cronje et al., 2012; Gest et al., 2012).

A novel systemic approach is promising in skipping difficulties possibly derived by single gene bio-engineering. Indeed it is emerging the concept that specific allelic forms of genes directly involved in ASC metabolism, or positively correlated with ASC storage, can differently affect ASC level in plant edible tissues. In particular, the identification of the QTL common or positively correlated to both ASC level and plant defense responses are opening new perspectives. In this context the comparison of wild progenitors and the derived cultivated lines seems to be very useful for identifying strategies adopted by plants during evolution for increasing their fitness. In a near future, these strategies could be implemented in crop by introgression through “classical” breeding techniques or multiple genes bio-engineering. In this perspective, the discovery of the main genetic mechanisms controlling ASC level in different plant species is a prerequisite that can allow scientists to identify successful strategies for vitamin C bio-fortification.

It is also necessary to move toward a novel and more efficient concept of biofortification, that at the same time takes into account the increase in nutritional value and plant stress tolerance as a unique goal of the improving strategy. Indeed, improvement of plant tolerance to adverse environmental conditions has also a direct positive effect on human health, as an example by possibly reducing the use of pesticides during field grown and post-harvest storage with an expectable effect on both environmental pollution and human health. Even if the level of pesticides are strictly controlled by law, a number of studies reports carcinogenic effects at high doses that can be also reaches for a low but prolonged exposure of toxic molecule (Alavanja and Bonner, 2012). Moreover, producing “fortified” crops in this novel perspective can also increase the availability of plant foods for the increasing world population. This is particularly challenging for reducing harvest and post-harvest crop losses and consequently food costs in an era of climatic changes increasing the geographic areas subjected to water and thermal stresses.

## Conflict of Interest Statement

The authors declare that the research was conducted in the absence of any commercial or financial relationships that could be construed as a potential conflict of interest.
